# A Pressure and Proximity Sensor Based on Laser-Induced Graphene

**DOI:** 10.3390/s24123907

**Published:** 2024-06-17

**Authors:** Jiatong Ye, Tiancong Zhao, Hangyu Zhang

**Affiliations:** 1 School of Biomedical Engineering, Faculty of Medicine, Dalian University of Technology, Dalian 116024, China; jiatongye@mail.dlut.edu.cn (J.Y.); zhaotiancong@163.com (T.Z.); 2 Liaoning Key Laboratory of Integrated Circuit and Biomedical Electronic System, Dalian University of Technology, Dalian 116024, China

**Keywords:** laser-induced graphene, oxidized graphene, pressure sensor, proximity sensor, three-dimensional porous dielectric layer

## Abstract

Smart wearable devices are extensively utilized across diverse domains due to their inherent advantages of flexibility, portability, and real-time monitoring. Among these, flexible sensors demonstrate exceptional pliability and malleability, making them a prominent focus in wearable electronics research. However, the implementation of flexible wearable sensors often entails intricate and time-consuming processes, leading to high costs, which hinder the advancement of the entire field. Here, we report a pressure and proximity sensor based on oxidized laser-induced graphene (oxidized LIG) as a dielectric layer sandwiched by patterned LIG electrodes, which is characterized by high speed and cost-effectiveness. It is found that in the low-frequency range of fewer than 0.1 kHz, the relative dielectric constant of the oxidized LIG layer reaches an order of magnitude of $10^{4}$. The pressure mode of this bimodal capacitive sensor is capable of detecting pressures within the range of 1.34 Pa to 800 Pa, with a response time of several hundred milliseconds. The proximity mode involves the application of stimulation using an acrylic probe, which demonstrates a detection range from 0.05 mm to 37.8 mm. Additionally, it has a rapid response time of approximately 100 ms, ensuring consistent signal variations throughout both the approach and withdrawal phases. The sensor fabrication method proposed in this project effectively minimizes expenses and accelerates the preparation cycle through precise control of laser processing parameters to shape the electrode-dielectric layer-electrode within a single substrate material. Based on their exceptional combined performance, our pressure and proximity sensors exhibit significant potential in practical applications such as motion monitoring and distance detection.

## 1. Introduction

With the increasing application of pressure sensors in wearable devices and electronic skin, flexible pressure sensors have attracted the interest of many researchers due to their outstanding flexibility and ductility [1,2,3,4,5]. Currently, flexible pressure sensors are categorized into resistance type, capacitance type, piezoelectric type, and triboelectric type based on their working principle [6,7,8]. Among these types, capacitive pressure sensors offer advantages such as simple structure, insensitivity to temperature and humidity, low power consumption, and wide applicability in human-computer interaction, medical health, robot haptics, and other fields [9,10,11]. However, these advantages also necessitate more stringent substrate material requirements [12,13]. For example, the material composing the device must be thin and soft. In certain situations, it should have the ability to conform to the surface of human skin or be implanted in the body [14]. This also requires that the material demonstrate excellent biocompatibility and achieve a strong mechanical match with biological tissues.

Recently, laser-induced graphene (LIG) has garnered significant attention as a promising material for flexible, high-performance, and cost-effective sensors [15,16,17]. The synthesis of LIG can be achieved using various carbon precursors, including sustainable resources such as trees [18], as well as high-performance polymers like polyimide (PI) [19], polyetherimide (PEI) [20], and phenolic resin (PR) [21]. Lasers induce a photothermal effect, in which the target material absorbs incident photon energy and converts it into thermal energy. The rapid deposition of this energy leads to the generation of exceptionally high temperatures, triggering processes such as carbonization, graphitization, and exfoliation. Ultimately, these processes culminate in the formation of three-dimensional (3D) porous structures characterized by remarkable surface area, scalability, and stability [22,23]. The proposed method facilitates the single-step in situ fabrication and patterning customization of flexible sensors [24,25], demonstrating exceptional sensitivity, remarkable biocompatibility, and robustness [26]. Moreover, the porous microstructure of LIG can significantly enhance the sensitivity of pressure sensors [27,28].

The potential of graphene as a revolutionary material for flexible wearable electronics has been widely acknowledged [29]. Furthermore, its exceptional properties render it an ideal two-dimensional filler in polymer nanocomposites [30]. The chemical properties of modified graphene oxide surpass those exhibited by pristine graphene. Graphene oxide (GO) has been extensively employed across various domains in recent years, including the development of high-performance nanocomposites, supercapacitors, and chemical sensors [31,32,33]. By manipulating easily adjustable processing parameters, laser irradiation enables the precise control of surface morphology, properties, chemical composition, and electrical characteristics in the production of LIGs. Plasma treatment of graphene-based materials facilitates the creation of edges, vacancies, distortions, and doping effects that enhance LIG’s porosity while imparting desired attributes to the material. This approach not only accelerates processing speed but also reduces costs [34,35]. The incorporation of heteroatoms during the fabrication process of LIG significantly enhances the performance of LIG-based devices. Although numerous studies have been conducted on the morphology, structure, properties, and applications of LIG, the preparation of flexible wearable devices using LIG remains a challenge from the perspective of parameter tuning and material selection during the preparation process.

Integrated multifunctional sensors represent a novel direction in sensor technology, characterized by their compact size, robust functionality, and centralized collection of information for easy processing [36]. The traditional human-machine interface primarily involves direct interaction between humans and machines through single-function pressure sensors. In contrast, proximity sensors, a type of non-contact sensor, are capable of detecting the presence of target objects without physical touch. This enables them to perform functions such as position recognition, obstacle avoidance, and human-machine interaction [37]. As the range of application scenarios continues to expand, there is an increasing demand for devices that can detect both contact and non-contact physical stimuli. Therefore, it is crucial to devise a simple and effective method for integrating capacitive pressure and proximity sensing capabilities into a single device in order to significantly enhance its functionality.

The present study introduces a bimodal sensor fabricated using oxidized LIG as the dielectric layer and LIG electrodes to enable seamless integration of pressure and proximity sensors. Moreover, through the optimization of electrode shape in the pressure sensor, it is possible to achieve proximity sensing with a broad detection range, enhanced sensitivity, and rapid response. Laser-induced graphene technology is utilized in the study to achieve facile and rapid patterning for the fabrication of compressible electrodes and dielectric layers. This facilitates the development of capacitive pressure sensors with exceptional precision and sensitivity.

## 2. Materials and Methods

### 2.1. The Sensor Fabrication

**Figure 1:**
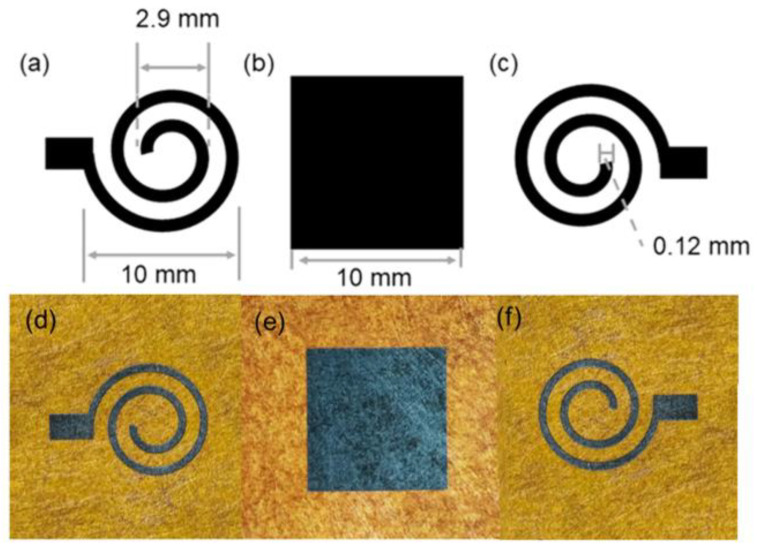
The design and photos of the electrodes and dielectric layer. (**a**) Dimensions and initial helix diameter of the Archimedes helix electrode peripherals. (**b**) Dimensions of the dielectric layer. (**c**) The width of the Archimedes spiral electrode. Photos of the LIG electrodes (**d**,**f**) and the dielectric layer (**e**).

Polyimide (PI) paper with a thickness of 90 µm is selected as the substrate material for LIG fabrication. LIG electrodes with the helix pattern (Figure 1a,c) are engraved on the PI paper using the same parameters as previously reported by a laser engraver (xTool LaserBox D1, Xtooltech Co., Ltd., Shenzhen, China) equipped with a 455 nm high-power (110 W) diode laser with a spot size of 80 µm [38]. The square dielectric layers (Figure 1b) are generated by engraving the PI paper and subsequent oxidation treatment on both sides. Specifically, the PI paper is engraved on both sides to form a continuous LIG tunnel through the PI paper, designated as through-LIG, which connects the upper and lower surfaces with an optimized power (13%), engraving speed (36 mm/s), and engraving density (300 Row/cm). The oxidation treatment is conducted using an oxygen plasma cleaner (Mingheng PDC-MG) with a two-way float flow meter range of 0.2–1.5 L/min. The pressure stimulation is applied by the 10 × 10 mm acrylic probe of the universal testing machine. Previous studies have demonstrated that sensors with fewer turns and lower widths exhibit higher relative capacitance change rates. Based on the optimized parameters, the upper and lower complementary spiral electrodes are fabricated in this experiment. The helix electrodes, with a starting helix diameter of 10 mm, a final helix diameter of 2.9 mm, several helix coils of 2, and a wire width of 0.12 mm, are formed by laser engraving on PI paper. The narrow width of the electrodes is designed to facilitate the distribution of electrons at the stripes, while the small number of turns is intended to reduce the blockage of the striped field. The dimensions and photos of the electrodes and the dielectric layer are illustrated in Figure 1. Double-sided conductive copper tape is affixed to the ends of two helix electrodes for connecting data acquisition equipment. Polyurethane (PU) tape is used to fix the layers and package the sensor.

### 2.2. Characterization Techniques and Performance Evaluation

The morphology of LIG electrodes and the elemental composition of LIG before or after the oxidation are examined by a TESCAN Mira3-LMH (TESCAN, Brno, Czech Republic) scanning electron microscope (SEM) with energy-dispersive X-ray spectroscopy (EDX). Raman analysis is performed using an Invia Qontor laser confocal microraman spectrometer (532 nm, 0.8 mW) manufactured by Renishaw PLC, Wotton-under-Edge, UK. X-ray photoelectron spectroscopy (XPS) measurements are performed with an ESCALAB XI+ X-ray photoelectron spectrometer from Thermo, Oxford, UK. The thin-layer resistance of the LIG electrode is measured using a four-probe impedance analyzer (RTS-9, 4-Probetech, 4 Probe Tech Co., Ltd., Guangzhou, China). The capacitance data for the sensor are collected at a frequency of 250 kHz using a TruEbox precision RC analyzer (TruEbox 01RC, LinkZill, Hangzhou, China). The mechanical pressure simulation in approach mode involved either a universal testing machine (ZQ-990B, Smart Precision Instrument, Okaya, Japan) or the manual placement of a homemade cardboard balance block near or away from the sensor probe. The relative permittivity at different frequencies is determined by measuring the capacitance at different frequencies using an electrochemical workstation (Zahner ZENNIUM X, Kronach, Germany).

## 3. Results and Discussion

### 3.1. Characterization of LIG

The thin layer resistance of LIG exhibits variability, which is contingent upon the dimensions and laser power [39,40]. The square through-LIG, measuring 10 × 10 mm in boundary size, is fabricated via laser engraving on both sides of PI paper. In the process of LIG fabrication, precise focusing of a laser beam can generate localized temperatures on the polyimide (PI) substrate that exceed the threshold for bond dissociation and facilitate carbon atom rearrangement into graphene. The simultaneous generation of gas products also leads to the establishment of a high-pressure environment, thereby facilitating the formation of LIG, characterized by its porous structure. When the laser power is low or the engraving speed is fast, insufficient energy absorption by the PI paper may hinder the complete carbonization of its internal components, resulting in a PI sandwich that fails to form a thorough LIG structure. Conversely, higher power levels promote increased graphitization and enhance film quality while reducing thin layer resistance; however, excessive power can also compromise the integrity of the LIG network, rendering it fragile and unstable. The effect of thermal power on graphitization has been demonstrated by Lin et al. [41]. By continuously optimizing the engraving parameters, the LIG electrode exhibits durability. The thin layer resistance of the single-sided LIG electrode is measured to be 1.33 ± 0.02 kΩ/sq.

**Figure 2:**
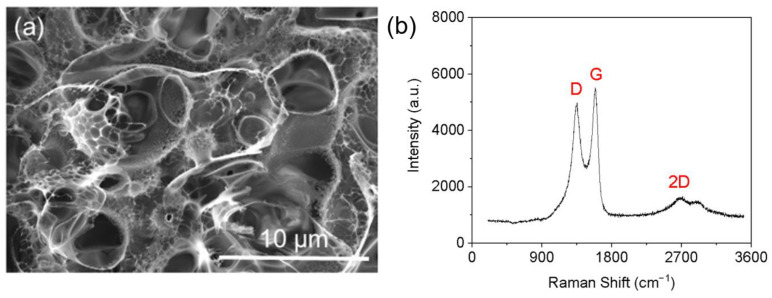
The SEM image (**a**) and Raman spectrum (**b**) of LIG.

The microstructure of LIG is characterized using SEM and confocal Raman microscopy. As shown in Figure 2a, the SEM image reveals a highly porous structure with varying pore sizes for LIG, which is attributed to the rapid formation of gaseous products during laser induction [42]. The Raman spectrum of LIG depicted in Figure 2b exhibits three prominent peaks that characterize LIG. Specifically, the D peak at approximately 1345 ${cm}^{-1}$ arises from defective or curved ${sp}^{2}$ carbon bonds; the G peak at approximately 1580 ${cm}^{-1}$ is linked to graphite-derived structures; the 2D peak at approximately 2700 ${cm}^{-1}$ is generated by laser processing-induced graphene structures. The degree of disorder in the carbon structure can be quantified and represented by the intensity ratio between G bands and D bands ($I_{D}$/$I_{G}$) [43]. The pronounced dominance of $I_{G}$ over $I_{D}$ peaks observed in the Raman spectra signify the exceptional crystallinity exhibited by graphene, which aligns with previous literature findings [44].

### 3.2. Characterization of Oxidized LIG Dielectric Layer

**Figure 3:**
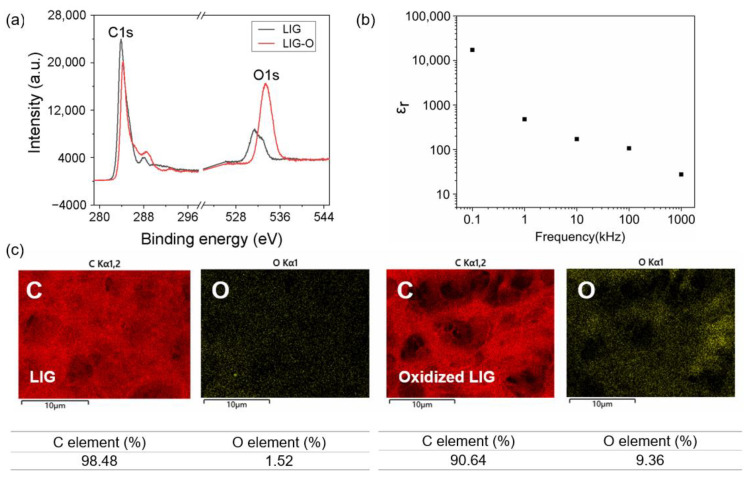
Materials characterization of oxidized LIG. (**a**) XPS of LIG and oxidized LIG. (**b**) Relative dielectric constants measured at different frequencies. (**c**) EDX analysis of the elemental content of LIG and oxidized LIG.

Oxidized LIG is fabricated through direct laser engraving on a PI substrate, followed by oxygen plasma treatment. It is well established that this process induces surface defects in carbon materials, introduces oxygen-containing groups, and alters the surface chemical properties [45,46]. Considering the slight error in laser operation over time, the power can be fine-tuned to create LIG with low conductivity. The subsequent stage entails the oxidation of LIG to yield oxidized LIG with an appropriate porous microstructure. XPS tests are conducted on LIG and oxidized LIG samples, and the obtained information regarding the surface functional groups is presented in Figure 3a. The dominant peak of the C1s core-level spectra for LIG appears at 284.5 eV, which is indicative of the presence of carbon atoms from the ${sp}^{2}$ graphene sheet [47]. However, noticeable discrepancies can be observed in the plasma spectra following functionalization. As depicted in Figure 3a, a substantial increase is evident in the peak value of O1s after plasma treatment, while the intensity of the C1s peak diminishes. This phenomenon is likely attributed to an augmented presence of oxygen-related chemical bonds after oxidation, such as oxygen-containing groups like C-OH and -COO [48,49,50]. The simultaneous occurrence of this phenomenon further substantiates the presence of oxygen-containing groups on LIG after oxidation [46].

The elemental composition of the oxidized LIG layer utilized in this study is further examined both before and after oxidation treatment. EDX elemental analysis is employed to investigate the elemental content of the oxidized LIG dielectric layer. The EDX test results presented in Figure 3c accurately quantified the change in oxygen content, which increased from 1.52% to 9.36%. This finding provides evidence that the oxygen content of the LIG dielectric layer increases following oxidation treatment using a plasma cleaner. Consequently, there is an overall increase in the degree of oxidation within the oxidized LIG layer, suggesting that it might be able to behave as a dielectric layer.

In addition, the resistances across the through-LIG before or after the oxidation are measured with samples prepared with different laser engraving parameters. The data presented in Table 1 reveal that by optimizing the laser engraving parameters, through-LIG undergoes a transformation from a conductive to an insulating layer after the same period of oxidation. This leads to changes in the electrical properties of LIG. This further validates our initial hypothesis that an insulated oxidized LIG dielectric layer can be achieved through oxidizing high-resistance LIG. The engraving parameter of sample 5 represents the stable critical condition for through-LIG insulation after oxidation. By affixing the oxidized LIG dielectric layer with a provided clamp in a vertical orientation, we are able to fabricate a typical parallel-plate capacitor for subsequent testing with a Zahner electrochemical workstation at different frequencies. The standard formula for calculating the dielectric constant of a dielectric material is as follows:

(1) $$\varepsilon_{r}=Cd/\varepsilon_{0}\cdot A,$$

where C represents the measured capacitance, d signifies the distance between two electrodes, $\varepsilon_{0}$ denotes the free space dielectric constant, and A indicates electrode area. The results of the relative dielectric constant measurements are displayed in Figure 3b. In the low-frequency range of less than 0.1 kHz, the oxidized LIG exhibits a high relative dielectric constant, which is comparable to that of the dielectric layer constructed with graphene oxide reported previously [51]. This reaffirms its high relative dielectric constant and supports its potential use as a high-dielectric material for capacitive energy storage.

### 3.3. Construction of Capacitive Pressure and Proximity Sensor

The effectiveness of capacitive proximity detection is contingent upon the coupled electric field, underscoring the importance of optimizing the structural design of the electrodes to enhance proximity sensing performance [52]. Previous studies have compared the impact of three electrode pattern designs (parallel plate, ring and disc, and helix) on device performance through simulation and experimental analysis. The findings concluded that employing complementary helix electrode patterns is one of the most effective methods for improving proximity sensing device performance [53]. To integrate pressure and proximity sensing functions, the upper and lower electrode patterns are designed as complementary helix structures.

**Figure 4:**
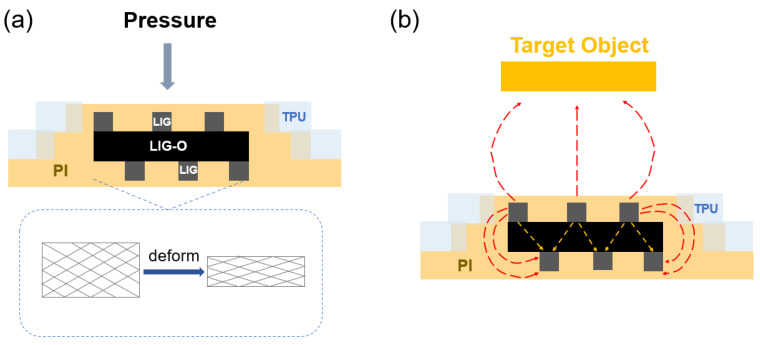
Working principle of the sensor in pressure mode (**a**) and proximity mode (**b**).

When the working area of the pressure mode sensor is subjected to external pressure, the capacitance value between the electrode plates will change due to the deformation of the middle oxidized LIG dielectric layer and the upper and lower electrodes, as shown in Figure 4a. In proximity mode, the sensor utilizes the edge electric field to detect approaching objects within the detectable range [54]. On the other hand, the mutual capacitance ($C_{mc}$) produced by the interaction between the outflowing electric field and the target object in the operational area of the proximity mode sensor varies according to the distance between the electrode and the target object [55]. The formula is as follows:

(2) $$C_{mc}=\frac{4\varepsilon w_{mc}}{\pi }ln\frac{2l_{mc}}{d_{mc}},$$

where ε represents the relative permittivity, $w_{mc}$ and $l_{mc}$ represent the width and length of the target object, respectively, and $d_{mc}$ represents the distance between the electrode and the target. The electric field lines in the vicinity of the electrode area of the proximity mode sensor are disrupted, and objects with different dielectric constants are incorporated into the electric field surrounding the device, resulting in alterations to the mutual capacitance of the device. The proximity to the device surface is a significant factor in the extent of the capacitance change; the closer the distance, the more pronounced the change. Conversely, the changes diminish with distance, as shown in Figure 4b.

### 3.4. Operational Performance of the Sensor

The pressure sensor operating curve contains two main types of information. The first is the response range, which refers to the range over which the device is artificially calibrated to respond to a signal while maintaining normal performance. The second type of information is sensitivity, which describes how the sensor responds to a unit strength of pressure by measuring the change in output signal relative to the magnitude of pressure change. This is calculated by dividing the change in output signal strength by the value of the pressure change. In general, a high sensitivity implies a high signal-to-noise ratio, resulting in an increased ability of the sensor to sense small pressure changes. The sensitivity of the pressure sensor is defined as follows:

(3) $$S=\frac{∆C/C_{0}}{∆P},$$

where S is the sensitivity, $C_{0}$ denotes the initial value of capacitance, ΔC denotes the capacitance change, and P is the applied external pressure.

**Figure 5:**
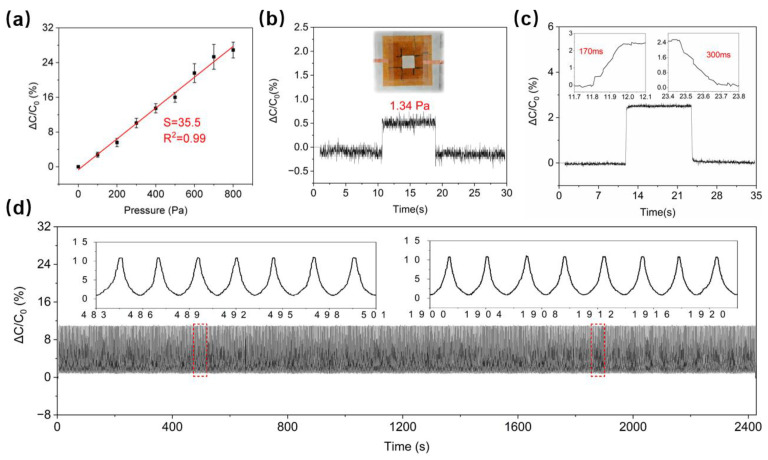
Pressure sensing performance. (**a**) Calibration curve of the pressure sensor. (**b**) Detection of the lower limit of the pressure sensor. (**c**) Response time and relaxation time of the pressure sensor. (**d**) Operational stability of the pressure sensor during 1000 loading and unloading cycles. The red dash frames indicate that these regions of signals are amplified for illustration.

A step pressure with a gradient of 100 Pa is applied to the flexible capacitive sensor by a tensile testing machine, and the capacitance change during compression of the sensor is measured by an output device. The step pressure response is illustrated in Figure S1. Our oxidized LIG pressure sensor exhibited higher sensitivity in the low-pressure range between 1.34 Pa and 800 Pa compared to previously reported sensors relying on metal electrodes and graphene series [11,56,57,58]. The linear sensitivity is found to be 35.5% kPa^−1^ with a linear regression coefficient of determination of 0.99 (Figure 5a).

The detection limit refers to the minimum pressure that the sensor can sense. In different application scenarios, the pressure detection range varies, so the lower sensing limit and sensing range are adjusted to maximize the sensitivity within the application range. To estimate the detection limit of this pressure sensor for dynamic forces, tiny mass weights with the same dimensions as the actual working size of the sensor and uniform mass distribution are prepared and placed vertically on the sensor with tweezers to record the sensor capacitance change. The minimum pressure detected by the sensor is determined to be 1.34 Pa, as illustrated in Figure 5b. The ultrasensitivity of this pressure sensor is attributed to the multilayer compressible microporous structure of oxidized LIG dielectric layers and LIG electrodes. Another advantage of this distinctive structure is the response time of the sensor, which is considerably shorter than that of the other metal- or graphene-based sensors. A pressure of 100 Pa was applied to the device using a universal testing machine, and the pressure was released once the signal stabilized, as shown in Figure 5c. The response time was measured at 170 ms, while the relaxation time was recorded at 300 ms.

Further experiments are carried out to comprehensively assess the pressure mode operating capability. A pressure of 300 Pa is cyclically applied and removed from the sensor 1000 times using a universal testing machine, the total durability test time is approximately 2450 s, and the changes in the sensor’s capacitance are recorded. The capacitance of the pressure sensor surface remained consistently stable throughout the cycle when subjected to 0.3 kPa of pressure 1000 times (Figure 5d). This indicates that the pressure sensor has a long service life and high reliability, further validating the consistent performance of the oxidized LIG-based pressure sensor.

**Figure 6:**
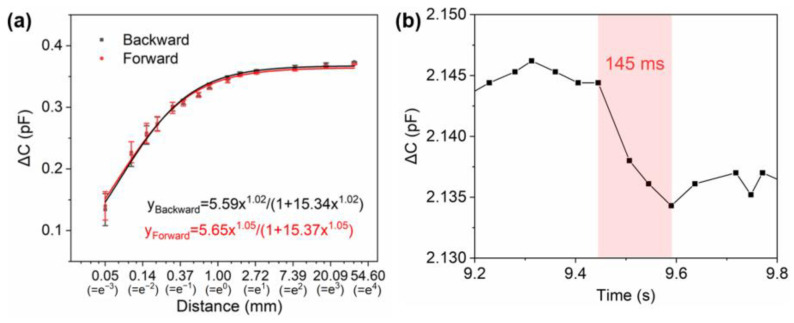
Operation performance of the proximity sensor. (**a**) The calibration curve of the proximity sensor. (**b**) The response time of the proximity sensor.

In the proximity sensing mode, the complementary spiral electrodes allow for the outflow of electric fields, which interact with the approximating of objects and respond capacitively to proximity. The capacitance value of an ideal shunt plate capacitor depends on the dielectric, relative area, and distance between the electrodes. Generally, it does not take into account the fringe effect. However, when considering the fringe field, the total capacitance can be divided into two parts: the classical capacitance equation ($C_{0}$) and the modified equation (∆C). For the square parallel plate capacitors:

(4) $$C=C_{0}+∆C=\frac{\varepsilon_{0}\times l\times \omega }{d}+\frac{\varepsilon_{0}\times \omega }{2\pi }ln\frac{2\pi \times l}{d},$$

where $\varepsilon_{0}$ represents the relative permittivity, l and ω represent the length and width of electrodes, respectively, and d represents the gap distance of the electrodes. When applied to the contactless proximity sensing mode, the classical part of the capacitance equation ($C_{0}$) remains a constant base value. However, the modified equation includes a component (∆C) that is dependent on the fringe field and mutual capacitance, and this component changes significantly as an object approaches. It can be observed from the equation that ∆C exhibits a logarithmic relationship with distance. Therefore, the horizontal coordinates of the curve representing distance-dependent capacitance change (C − $C_{0}$) are represented logarithmically, as shown in Figure 6a. It is observed that the sensor’s sensitivity to the distance change exhibited a decline with the increase in distance between the probe and the surface of the device. Consequently, the operating curve displays a nonlinear nature. To more accurately reflect the performance of the sensor across its full operational range, the LangmuirEXT1 power function is employed for fitting. The close and faraway results exhibit a high fitting accuracy, with an $R^{2}$ of 0.99, and the power function fitting results are also highly correlated. The specific equations are presented in Figure 6a. The fitted LangmuirEXT1 equation includes the sensitivity information. This result aligns with Equation (4), indicating a power-law relationship between the coupling capacitance and the approach distance. Given the close relationship between the coupling capacitance and the device capacitance, it can be reasonably assumed that the relationship between the sensor device capacitance and distance also adheres to a power law. A series of distance stimuli are applied at varying distance stages using an acrylic probe. This results in an operational range for the device between 0.05 mm and 37.8 mm. The press probe is positioned as closely as possible to the working area directly above, with a distance stimulus of 0.05 mm and a moving speed of 100 mm/s. The duration from the commencement of the test until the stabilization of the capacitance signal, referred to as the sensor response time, is depicted in Figure 6b.

### 3.5. Applications of the Sensor

**Figure 7:**
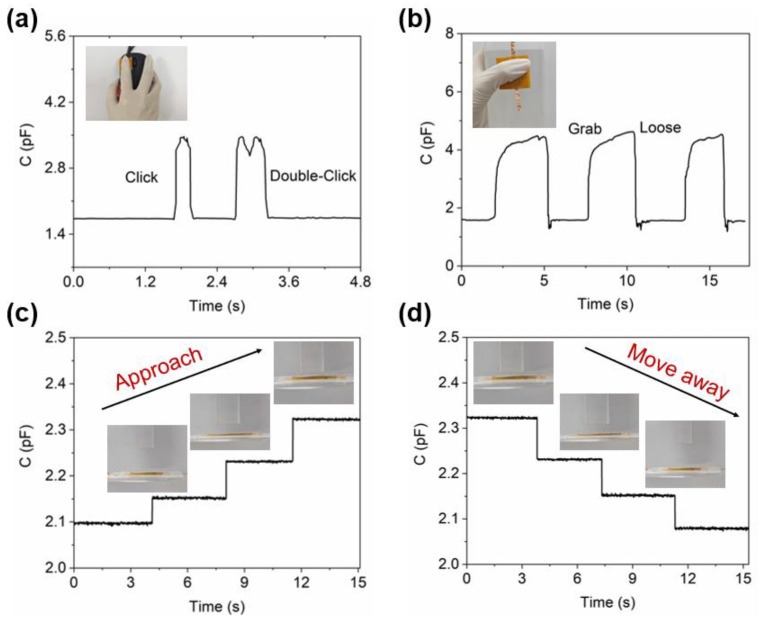
The practical application of the sensor. (**a**) Quick click tests under pressure mode. (**b**) Grip and release tests under pressure mode. Distance tests in proximity mode when the object is approaching (**c**) or moving away (**d**).

The outstanding mechanical properties of LIG-based materials render the sensors with LIG electrodes and oxidized LIG dielectric layers suitable for a wide range of applications. The accurate estimation of contact information in the contact sensing mode is essential for identifying the properties of objects, such as manipulators. In this study, the responsiveness of the sensors is assessed under two different pressure states: mouse click and palm grip. In the test involving the mouse button application, it was observed that the sensor’s signal changed in response to single and double-click actions performed by the human hand’s fingertip. The click action takes approximately 240 ms to complete, and the resulting signal is reflected as a single peak. While the double-click action with similar force is completed within 390 ms, the signal is also accurate and produces precise and rapid changes, as shown in Figure 7a. The experimental results demonstrate that the oxidized LIG-based flexible pressure sensor can be effectively applied in scenarios requiring short response times, such as hydraulic braking systems in vehicles like automobiles. Short, fast response pressure monitoring is necessary to ensure the quick generation of sufficient braking force during emergency braking and to prevent brake failure. Furthermore, the sensor is employed for detecting repeated grip-release of a water bottle, exhibiting stable and synchronous responses (Figure 7b). Upon attaining a stable grasping action, the signal gradually changes with slight variations in strength due to human operation factors. The experimental findings also demonstrate that the oxidized LIG-based flexible pressure sensor can be effectively utilized for long-term scenarios and reliably responds to demand, such as in industrial production processes. Long-term stable pressure signal monitoring can assist enterprises in real-time production process monitoring, timely adjustment, and preventive maintenance.

Furthermore, the sensor under investigation incorporates an approach mode characterized by the responsiveness of the device signal to alterations in the distance between the object and the sensor. The complementary spiral electrode pattern allows the sensor to possess proximity-sensing properties. With a gradient of 0.01 N, the pressure probe is controlled to apply pressure, and the press controls the probe stroke through feedback. When the object gradually approaches the sensor at a fixed distance, the capacitive response of the sensor exhibits a step-increase trend, as depicted in Figure 7c. Conversely, when the object gradually moves away from the sensor, the capacitive response of the sensor demonstrates a step-decrease trend (Figure 7d). During this process, the capacitive response caused by the same distance change remains constant, indicating excellent stability of the device. Through the application test of the “upper step” and “lower step” proximity principles on target objects, sensing the proximity of the target object can enable location recognition, obstacle avoidance, human-computer interaction, and other functions with broad potential applications. Future research should focus on optimizing the sensor structure to enhance its accuracy and stability and promote its extensive application and development in the field of intelligence.

## 4. Conclusions

In summary, a capacitive flexible pressure and proximity sensor has been developed utilizing LIG electrodes and oxidized LIG dielectric layers. This sensor offers several advantages, including high sensitivity, fast response, and a simple structure. To achieve this, the resistance of LIG is tailored by optimizing the parameters of laser engraving and plasma treatment to create an insulating dielectric layer with a three-dimensional structure and mechanical elasticity. This modified LIG plays a critical role in the sensor’s overall structure and performance. Experimental testing has confirmed the suitability of the oxidized LIG, as proposed in this project, for use as a dielectric layer. Our experiments have shown that the sensors perform well in both contact and proximity modes, indicating their superior capability in real-world applications. We hope that future research in this field will build upon these findings to realize a broader deployment of dual-mode sensors.

## Figures and Tables

**Table 1:** The resistances across the through-LIG before or after the oxidation.

	Sample 1	Sample 2	Sample 3	Sample 4	Sample 5	Sample 6
LIG	3.4 kΩ	5.75 kΩ	6.5 kΩ	0.135 MΩ	1.2 MΩ	11 MΩ
Oxidized LIG	14.3 kΩ	78 kΩ	3.9 MΩ	Unstable	\	\

## Data Availability

The original contributions presented in the study are included in the article, and further inquiries can be directed to the corresponding author.

## Supplementary Materials

The following supporting information can be downloaded at: https://www.mdpi.com/article/10.3390/s24123907/s1, Figure S1. Dynamic response and recovery curves under different pressures.
