# Supplementary material for: A Pressure and Proximity Sensor Based on Laser-Induced Graphene
Source: Sensors (Basel). 2024 Jun 17;24(12):3907. doi: 10.3390/s24123907 (PMC11207858; doi:10.3390/s24123907)
Supplement: Supplementary file 1 [file sensors-24-03907-s001.zip › sensors-3026754-supplementary.pdf]

Supporting information

A pressure and proximity sensor based on laser-induced graphene

Jiatong Ye[1], Tiancong Zhao[1], Hangyu Zhang[1,2,*]

[1] School of Biomedical Engineering, Faculty of Medicine, Dalian University of Technology, Dalian 116024, China

[2] Liaoning Key Lab of Integrated Circuit and Biomedical Electronic System, Dalian University of Technology, Dalian 116024, China

***** Corresponding author

Email: hangyuz@dlut.edu.cn

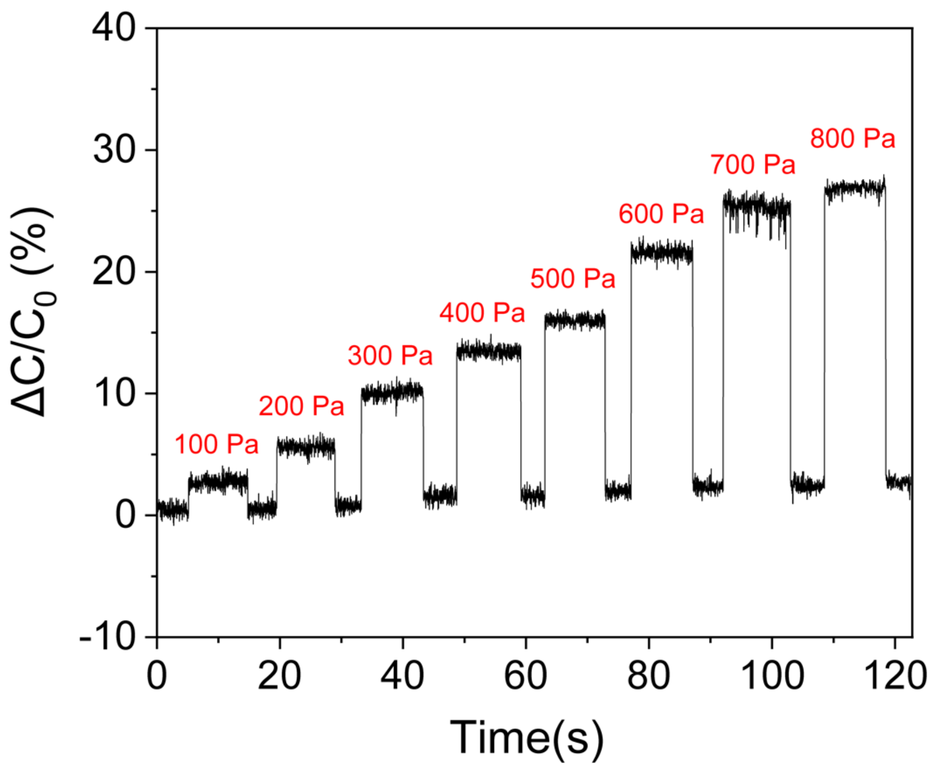

Figure S1. Dynamic response and recovery curves under different pressures.
